# [^11^C]Martinostat PET analysis reveals reduced HDAC I availability in Alzheimer’s disease

**DOI:** 10.1038/s41467-022-30653-5

**Published:** 2022-07-19

**Authors:** Tharick A. Pascoal, Mira Chamoun, Elad Lax, Hsiao-Ying Wey, Monica Shin, Kok Pin Ng, Min Su Kang, Sulantha Mathotaarachchi, Andrea L. Benedet, Joseph Therriault, Firoza Z. Lussier, Frederick A. Schroeder, Jonathan M. DuBois, Baileigh G. Hightower, Tonya M. Gilbert, Nicole R. Zürcher, Changning Wang, Robert Hopewell, Mallar Chakravarty, Melissa Savard, Emilie Thomas, Sara Mohaddes, Sarah Farzin, Alyssa Salaciak, Stephanie Tullo, A. Claudio Cuello, Jean-Paul Soucy, Gassan Massarweh, Heungsun Hwang, Eliane Kobayashi, Bradley T. Hyman, Bradford C. Dickerson, Marie-Christine Guiot, Moshe Szyf, Serge Gauthier, Jacob M. Hooker, Pedro Rosa-Neto

**Affiliations:** 1 Translational Neuroimaging Laboratory, Department of Neurology and Neurosurgery, Faculty of Medicine, The McGill University Research Centre for Studies in Aging, McGill University, Montreal, QC Canada; 2 Departments of Psychiatry and Neurology, University of Pittsburgh School of Medicine, Pittsburgh, PA USA; 3 Departments of Neurology, University of Pittsburgh School of Medicine, Pittsburgh, PA USA; 4 Montreal Neurological Institute, McGill University, Montreal, QC Canada; 5 Department of Molecular Biology, Ariel University, Ariel, Israel; 6 Department of Pharmacology and Therapeutics, McGill University, Montreal, QC Canada; 7 Neurology Athinoula A. Martinos Center for Biomedical Imaging, Department of Radiology, Massachusetts General Hospital, Harvard Medical School, Charlestown, MA USA; 8 Departments of Biological and Biomedical Engineering and Psychiatry, Douglas Mental Health University Institute, Brain Imaging Centre, Montreal, QC Canada; 9 Department of Psychology, McGill University, Montreal, QC Canada; 10 Department of Neurology, Massachusetts General Hospital, Harvard Medical School, Boston, MA USA

**Keywords:** Diagnostic markers, Alzheimer's disease

## Abstract

Alzheimer’s disease (AD) is characterized by the brain accumulation of amyloid-β and tau proteins. A growing body of literature suggests that epigenetic dysregulations play a role in the interplay of hallmark proteinopathies with neurodegeneration and cognitive impairment. Here, we aim to characterize an epigenetic dysregulation associated with the brain deposition of amyloid-β and tau proteins. Using positron emission tomography (PET) tracers selective for amyloid-β, tau, and class I histone deacetylase (HDAC I isoforms 1–3), we find that HDAC I levels are reduced in patients with AD. HDAC I PET reduction is associated with elevated amyloid-β PET and tau PET concentrations. Notably, HDAC I reduction mediates the deleterious effects of amyloid-β and tau on brain atrophy and cognitive impairment. HDAC I PET reduction is associated with 2-year longitudinal neurodegeneration and cognitive decline. We also find HDAC I reduction in the postmortem brain tissue of patients with AD and in a transgenic rat model expressing human amyloid-β plus tau pathology in the same brain regions identified in vivo using PET. These observations highlight HDAC I reduction as an element associated with AD pathophysiology.

## Abstract (web summary)

The link between amyloid and tau proteins with Alzheimer’s disease progression remains unclear. Here, the authors propose HDACs I downregulation as an element linking the deleterious effects of brain proteinopathies with disease progression.

## Introduction

Alzheimer’s disease (AD) is pathologically characterized by the brain deposition of amyloid-β and tau proteins^1^. However, how these proteinopathies interact to determine downstream neurodegeneration and cognitive impairment is poorly understood. It has been proposed that the progressive accumulation of protein aggregates imposes neuroepigenetic modifications to brain tissue determining the patient’s vulnerability to dementia^2,3^. Epigenetic regulation of gene expression is essential for maintaining mammalian cognitive function^4^. Among the epigenetic regulators, histone acetylation has been the most frequently associated with AD in recent studies^2,3^. Increased histone acetylation due to a reduction of class I histone deacetylases (HDACs I) has been shown to improve learning and memory in rodents^5–10^. Based on these observations, increased HDACs I level has been hypothesized to impair cognition in neurodegenerative conditions^7,10,11^. Indeed, studies show HDAC I increase in selected brain regions in animal models of AD and human postmortem tissue^2,12–16^, and propose that cognitive decline in AD is linked to increased HDAC I^2,3,6,7,17–20^. These results have been used as justification for clinical trials testing HDAC I inhibitors to improve cognitive symptoms in patients with AD^2,17,18,21^. On the other hand, recent postmortem series suggest decreased HDAC I levels in AD and caution with the use of HDAC I inhibitors in living people^22,23^.

Here, using a molecular imaging agent selective for HDACs I (isoforms 1–3) quantified across the whole brain^24^, we show that HDACs I availability is reduced in living patients with AD in brain regions vulnerable to both amyloid-β and tau pathologies. Moreover, structural equation modeling reveals that HDACs I reduction mediates the effects of both amyloid-β and tau on brain atrophy and cognitive impairment. Subsequent post-mortem analysis confirms that the HDAC I isoforms 1, 2, and 3 are reduced in patients with AD within vulnerable brain regions identified with in vivo imaging. Using transgenic rats, we find HDAC1 and HDAC2 reductions in animals expressing human amyloid-β and tau pathology, contrasting with normal levels in rats expressing single human amyloid-β pathology.

## Results

Ninety-four individuals (25 cognitively unimpaired (CU) young, 28 CU elderly, 15 MCI, and 26 AD dementia) were studied with [^11^C]Martinostat PET, MRI, and cognitive assessments; a subset had amyloid-β PET and tau PET. There was no significant difference in age between CU elderly, MCI, and AD dementia individuals (*P* = 0.0713). Demographic characteristics of the population are presented in Table 1. We assessed postmortem brain tissue of 15 individuals (6 AD dementia and 9 CU elderly) and two transgenic rat models (McGill-R-Thy1-APP and TgF344-AD).

**Table 1:** Demographics of the participants in each study site.

	CU young		CU elderly		MCI	AD	
	**MCSA**	**MGH**	**MCSA**	**MGH**	**MCSA**	**MCSA**	**MGH**
Number (*n*)	2	23	15	13	15	16	10
Age, yr, mean (SD)	23 (1.4)	27.2 (5.3)	67.4 (8.3)	63.2 (7.8)	72 (7)	69.7 (12)	66.9 (8.9)
Male, no. (%)	1 (50)	11 (48)	6 (40)	7 (54)	9 (60)	9 (56)	8 (80)
*APOE ε4*, no. (%)	0 (0)	–	5 (33)	–	4 (27)	8 (50)	–
Education, yr, mean (SD)	17.5 (0.7)	–	17.4 (4)	–	15.2 (4.8)	14 (4.6)	–
MMSE score, mean (SD)	30 (0)	–	29.5 (0.5)	29.1 (0.9)	27 (2.3)	14.4 (6.8)	19.9 (6.2)
CDR score, mean (SD)	0 (0)	–	0 (0)	0 (0)	0.5 (0)	1.4 (0.5)	0.8 (0.34)
[^18^F]AZD4694 SUVR, mean (SD)	1.39 (0.2)	–	1.53 (0.3)	–	2 (0.5)	2.9 (0.6)	–
[^18^F]AZD4694+, no. (%)	0 (0)	–	4 (27)	–	11 (73)	16 (100)	–
[^18^F]MK-6240 SUVR, mean (SD)	0.97(0.15)	–	1.1 (0.16)	–	1.7 (.8)	3.6 (1.1)	–
[^18^F]MK-6240+, no. (%)	0 (0)	–	3 (20)	–	11 (73)	16 (100)	–
GM density, mean (SD)	0.7(0.002)	–	0.64(0.03)	–	0.59(0.05)	0.48(0.05)	–
GM+, no. (%)	0 (0)	–	2 (13)	–	7 (47)	16 (100)	–

Demographic breakdown by study site.

*MCSA* McGill University Research Centre for Studies in Aging, *MGH* Massachusetts General Hospital, Harvard Medical School, *GM* Grey matter.

### [^11^C]Martinostat SUVR in vivo measurement of HDAC I availability

**Fig. 1:**
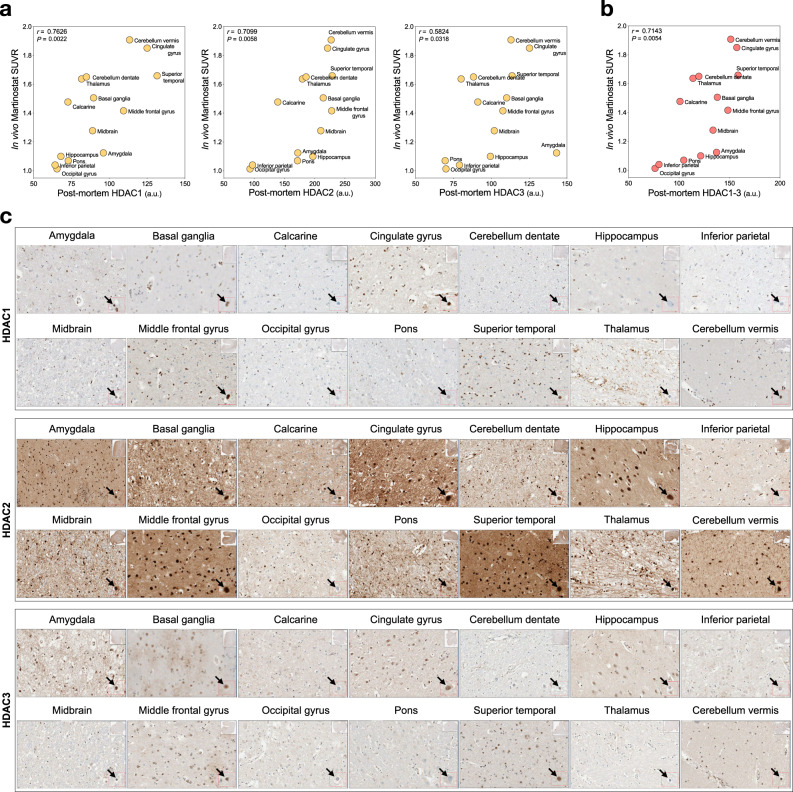
In vivo [^11^C]Martinostat SUVR correlates with postmortem HDAC1–3 levels. Scatter plots show two-sided Spearman’s correlation between in vivo [^11^C]Martinostat SUVR and postmortem nuclear **a** HDAC1–3 and **b** their averaged values in corresponding brain regions in an AD participant who underwent a [^11^C]Martinostat scan before death. **c** The panels show immunohistochemical stains for HDAC1, HDAC2, and HDAC3 across all the studied brain regions in the patient with AD. The arrows show magnified representative neuron nucleus from where HDACs levels were measured. a.u. arbitrary unit. Source data are provided as a Source Data file.

We performed an in vivo-postmortem study in a brain donor who underwent a [^11^C]Martinostat scan 22 months before death. We found that regional in vivo [^11^C]Martinostat SUVR was correlated with postmortem nuclear HDAC1–3 levels in neurons located in the corresponding brain regions (Fig. 1). Also, we studied the topographical similarity between [^11^C]Martinostat uptake in AD and HDAC1–3 gene expressions obtained from the Allen Human Brain Atlas. We found that in vivo regional mean [^11^C]Martinostat SUVR was highly correlated with Allen HDAC1-3 mRNA expression in the corresponding brain regions (Supplementary Fig. 1).

### [^11^C]Martinostat uptake is reduced in Alzheimer’s disease

**Fig. 2:**
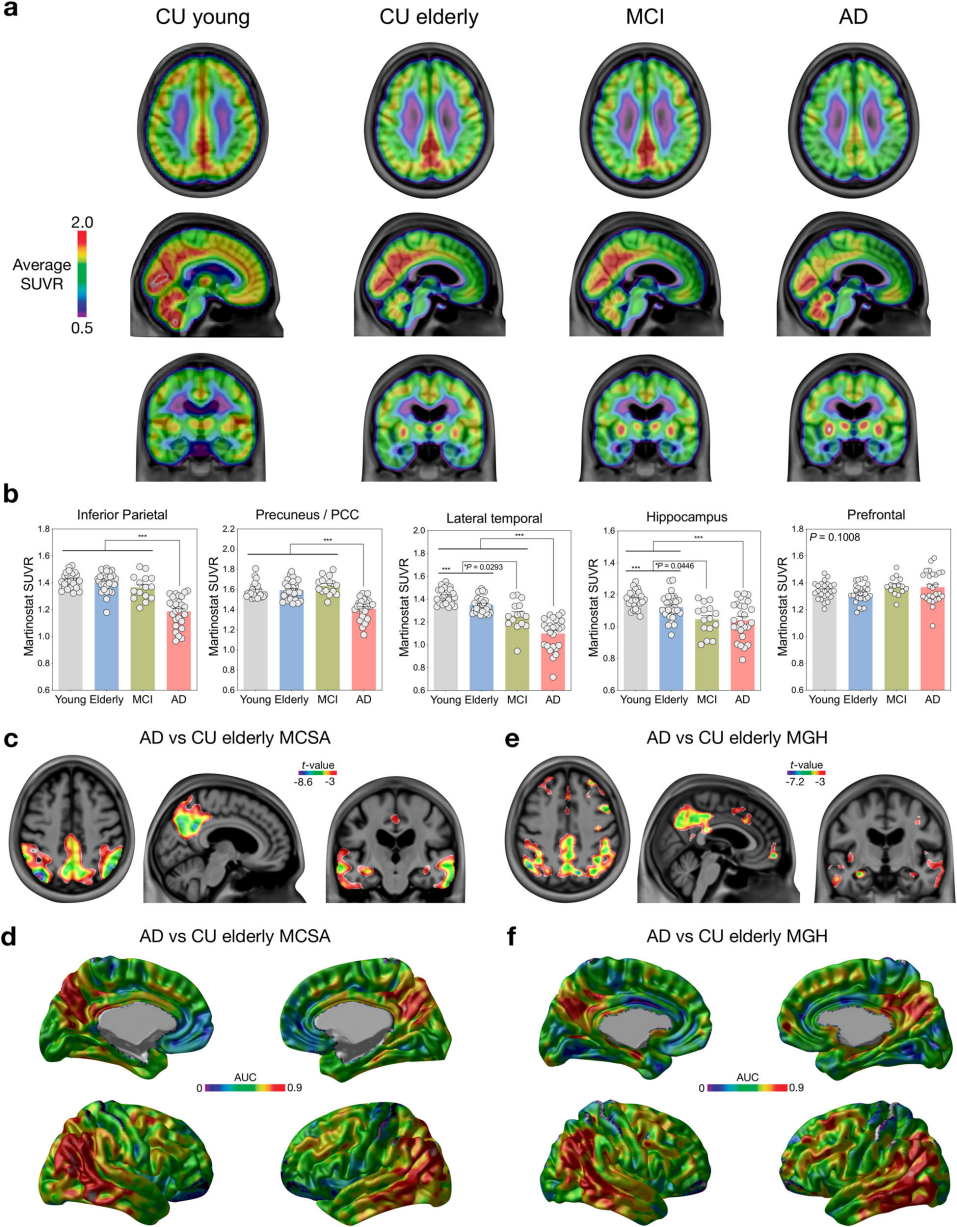
[^11^C]Martinostat SUVR is reduced in Alzheimer’s disease. **a** [^11^C]Martinostat SUVR images averaged among the entire population of CU young (*n* = 25) and elderly (*n* = 28), MCI (*n* = 15), and AD (*n* = 26) participants. [^11^C]Martinostat uptake was visually reduced in patients with AD in the posterior cingulate, precuneus, inferior parietal, and lateral temporal cortices. **b** Analysis of variance with Tukey’s multiple comparison among CU young and elderly, MCI, and AD dementia participants reveals that [^11^C]Martinostat uptake was reduced in AD dementia patients in the inferior parietal, posterior cingulate, precuneus, and lateral temporal cortices and reduced in MCI individuals in the inferior parietal and hippocampal cortices. Values are mean ± standard error of the mean and *** indicates *P* values < 0.001. Source data are provided as a Source Data file. **c** *T*-statistical parametric map (false discovery rate corrected for multiple comparisons at *P* < 0.05) shows the regions where [^11^C]Martinostat SUVR was significantly reduced in patients with AD (*n* = 16) compared to CU elderly (*n* = 15) at the MCSA site. **d** Voxel-wise receiver operating characteristic curve analysis conducted in the MCSA individuals reveals that [^11^C]Martinostat reduction discriminated AD from CU elderly with a high area under the curve (AUC) in large clusters in the posterior cingulate, precuneus, inferior parietal, and lateral temporal cortices. **e** and **f** The replication study conducted at the MGH site confirmed [^11^C]Martinostat uptake reduction in patients with AD (*n* = 10) compared to CU elderly (*n* = 13) in the same set of brain regions found in the MCSA cohort (false discovery rate corrected at *P* < 0.05). There was no significant increase in [^11^C]Martinostat uptake in patients with AD in both enrollment sites.

**Fig. 3:**
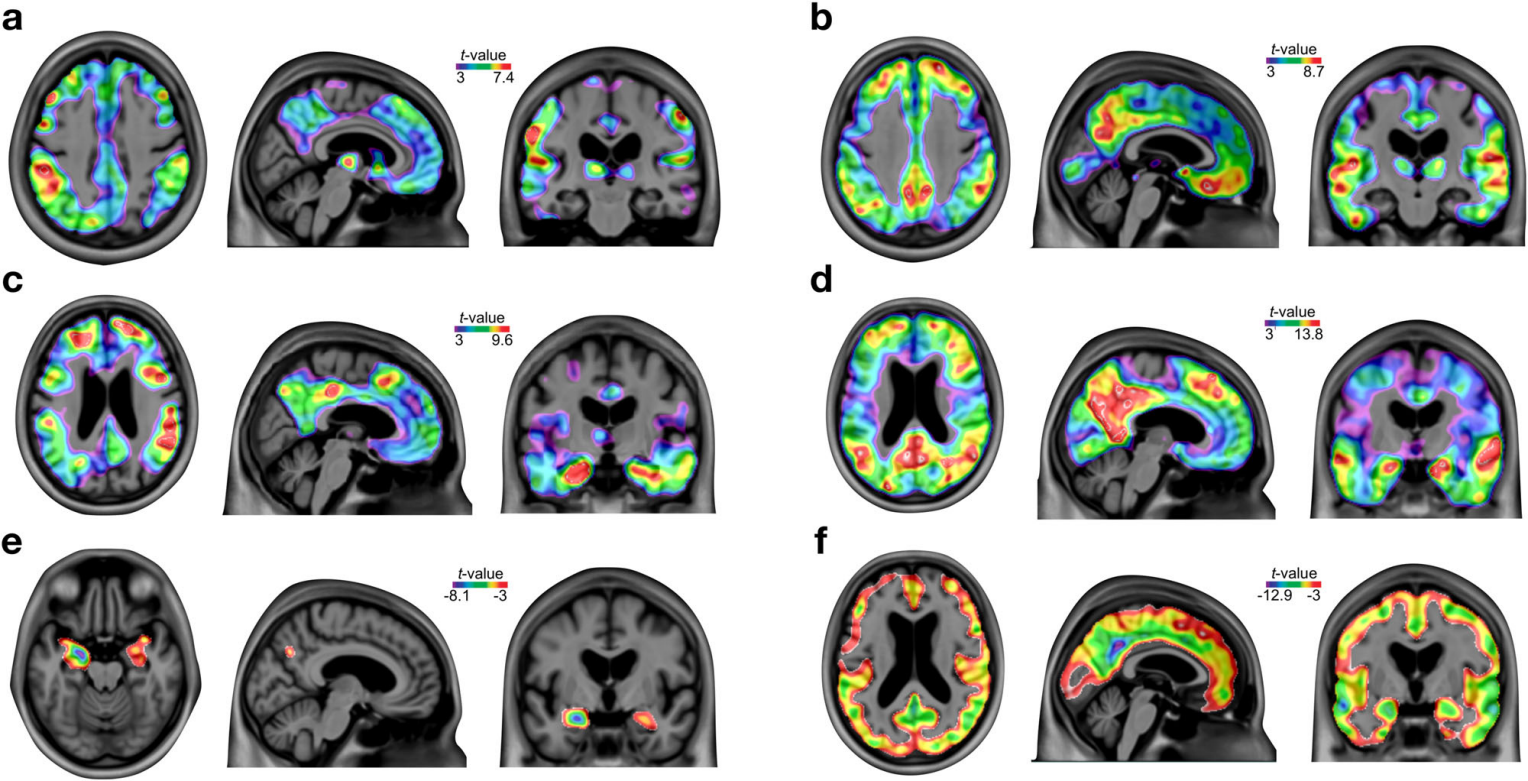
Regional diagnostic effects on amyloid-β, tau, and gray matter density. *T*-statistical parametric maps of two-sided *t*-tests false discovery rate corrected for multiple comparison at *P* < 0.05 show increased amyloid-β PET in **a** MCI (*n* = 14) and **b** patients with AD (*n* = 15); tau PET in **c** MCI (*n* = 14) and **d** patients with AD (*n* = 15); and decreased gray matter density in **e** MCI (*n* = 15) and **f** patients with AD (*n* = 16) as compared to CU elderly, in individuals at the MCSA site.

**Fig. 4:**
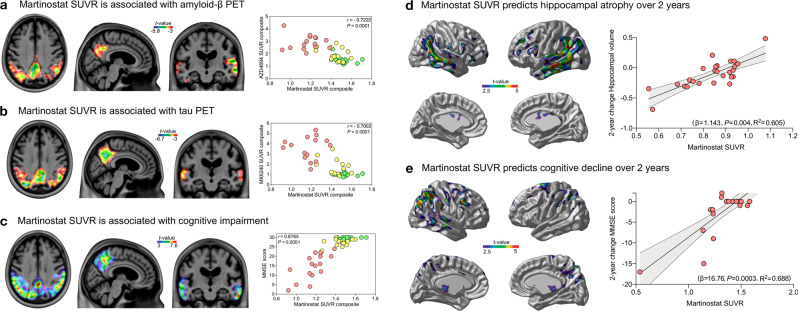
[^11^C]Martinostat SUVR is associated with Alzheimer’s disease pathophysiology and predicts longitudinal neurodegeneration and cognitive decline. *T*-statistical parametric maps show the results of voxel-wise linear regressions (left side panel) and scatter plots (right side panel) the results of Pearson correlations in regions showing [^11^C]Martinostat reduction in AD (Fig. 2c) between [^11^C]Martinostat PET and: **a** amyloid-β PET (*n* = 45). **b** tau PET (*n* = 44). **c** cognition measured with MMSE score (*n* = 48). [^11^C]Martinostat SUVR was associated with AD pathophysiology in the posterior cingulate, precuneus, inferior parietal, and lateral temporal cortices but not in other brain regions. Green, yellow, and red dots represent CU, MCI, and AD individuals who underwent amyloid-β or tau PET scans at the MCSA site, respectively. Linear regressions voxel-wise (left side panel) and in scatter plots within significant brain regions (right side panel) show that [^11^C]Martinostat SUVR predicts 2-year **d** hippocampal atrophy (*n* = 26) and **e** cognitive decline (*n* = 23) accounting for amyloid-β, tau, atrophy, age, and sex. The error bands represent a 95% Confidence Interval. For changes in hippocampal volume and MMSE score, lower scores represent a decrease in brain volume and cognitive decline, respectively. All the *t*-statistical maps were false discovery rate corrected for multiple comparisons at *P* < 0.05. Source data are provided as a Source Data file.

We found [^11^C]Martinostat SUVR reduction in AD in the posterior cingulate, precuneus, inferior parietal, lateral temporal, and hippocampal cortices compared with CU elderly (Fig. 2a–d). These results were independently replicated at a separate enrollment site without previous knowledge of these outcomes (Fig. 2e, f). There were no regions with a significant increase in [^11^C]Martinostat SUVR in patients with AD after multiple comparison corrections in both sites. These results were not driven by brain atrophy or changes in perfusion (Supplementary Figs. 2, 3, and 4a). As expected, individuals with MCI and AD dementia had increased amyloid-β PET and tau PET burden as well as brain atrophy (Fig. 3). In the posterior cingulate, precuneus, inferior parietal, and lateral temporal cortices, [^11^C]Martinostat SUVR was highly negatively correlated with brain amyloid-β and tau concentrations (Fig. 4a, b), and highly positively correlated with cognitive performance (Fig. 4c). [^11^C]Martinostat SUVR predicted 2-year longitudinal hippocampus atrophy (Fig. 4d) and cognitive decline (Fig. 4e) independently of baseline amyloid-β, tau, and atrophy levels (Table 2).

**Table 2:** [^11^C]Martinostat SUVR predicts longitudinal hippocampus atrophy and cognitive decline.

Variable	Beta	Std. error	*P* value
ΔHV ~ Martinostat + amyloid-β + tau + atrophy + age + sex			
Martinostat PET	1.143412	0.34851	0.00393
Amyloid-β PET	−0.034534	0.07611	0.65516
Tau PET	−0.050966	0.043763	0.2586
Atrophy (VBM)	−0.228354	0.726427	0.75668
Age (years)	−0.003158	0.003612	0.39286
Sex	−0.04022	0.065566	0.54687
ΔMMSE ~ Martinostat + amyloid-β + tau + atrophy + age + sex			
Martinostat PET	16.757979	3.596115	0.000261
Amyloid-β PET	0.420176	1.797558	0.818145
Tau PET	−0.901257	0.997334	0.379578
Atrophy (VBM)	7.972514	17.065449	0.646675
Age	0.005431	0.083415	0.948896
Sex	−1.48183	1.52421	0.345423

The table shows the results of beta, standard error, and *P* value coefficients of the linear regression models presented in Fig. 4d, e. [^11^C]Martinostat SUVR predicted a 1-year decrease in hippocampal volume (HV) (neurodegeneration) and worsening in mini-mental state examination (MMSE) scores (cognitive decline) in a subset of our population (*n* = 26; 13 CU, 7 MCI, 6 AD dementia), accounting for amyloid-β PET SUVR, tau PET SUVR, atrophy (VBM), age (years) and sex (female = 1). The imaging biomarker values were extracted from the brain regions showing [^11^C]Martinostat reduction in AD (Fig. 2c). For changes (Δ) in HV and MMSE score, lower scores represent a decrease in brain volume and cognitive decline, respectively.

### [^11^C]Martinostat uptake mediates atrophy and cognitive impairment

**Fig. 5:**
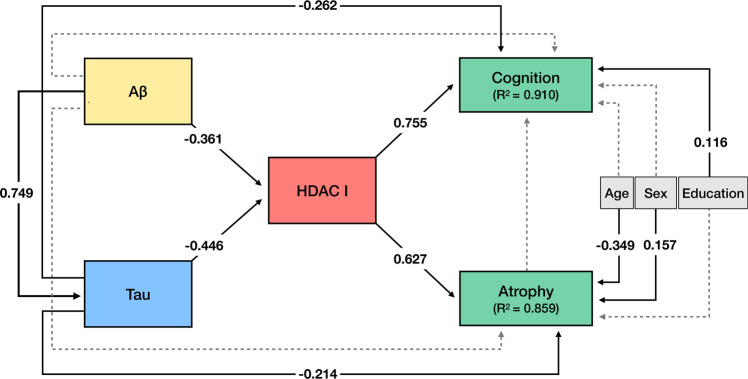
HDAC I mediates the effects of amyloid-β and tau pathology on cognitive impairment. The figure shows the structural equation model estimates of the associations between brain amyloid-β PET, tau PET, HDAC I PET, atrophy, as well as cognition. Solid lines represent significant effects, whereas dashed lines represent non-significant effects. The effect sizes (β estimates) presented in the figure are standardized and therefore may be compared. The model fits the data well (*n* = 44, *X*^2^ = 1.818, degrees of freedom = 6, *P* = 0.936, root mean squared error of approximation = 0.000 (90% confidence interval [0.000–0.049]), standardized root mean square residual = 0.029, comparative fit index = 1.000, Akaike information criterion = 871.632, and Bayesian information criterion = 907.316). The model revealed that HDAC I reduction mediates the deleterious effects of amyloid-β and tau on cognitive impairment and atrophy. This model explained 85.9% of the atrophy variance and 92% of the cognitive impairment variance. Tau also showed a significant direct effect on cognitive impairment and atrophy. Older age and sex (male) were associated with atrophy, whereas lower education was associated with worse cognition. The model inferred no direct association between atrophy and cognition, suggesting that their correlation occurs since the same pathological pathways affect both. The imaging biomarker values were extracted from regions showing [^11^C]Martinostat reduction in AD (Fig. 2c). PET biomarker values were adjusted for age. Cognition and atrophy were measured with MMSE and gray matter density, respectively.

We investigated whether HDAC I level measured with [^11^C]Martinostat SUVR mediates the deleterious effects of amyloid-β and tau on brain atrophy and cognitive impairment using a structural equation model. The model showed that [^11^C]Martinostat SUVR mediated the effects of amyloid-β. Similarly, [^11^C]Martinostat SUVR partially mediated the effect of tau on brain atrophy and cognitive impairment, yielding mediation effects size of 57% and 51%, respectively (Fig. 5). This construct was able to explain 85.9% and 91% of the variance in atrophy and cognitive impairment, respectively. Other constructs using the aforementioned markers such as models where HDAC I reduction precedes amyloid-β and tau pathology or a model where HDAC I reduction succeeds atrophy fitted the data poorly (Supplementary Fig. 4 and Supplementary Tables 2–4). The model testing the classical sequential model of AD progression without using HDAC I explained 66.8% and 80.1% of atrophy and cognitive impairment variance, respectively (Supplementary Fig. 5 and Supplementary Table 5). The models’ goodness-of-fit parameters are summarized in Table 3.

**Table 3:** Models’ goodness-of-fit.

Parameter	Model 1	Model 2	Model 3	Model 4	Model 5
RMSEA	0.000*	0.244	0.389	0.387	0.056
SRMR	0.029*	0.123	0.090	0.089	0.037
CFI	1.00*	0.935	0.724	0.726	0.995
AIC	871.632*	895.476	942.515	941.764	998.189
BIC	907.316*	931.159	971.062	970.311	1021.383

The construct suggesting that [^11^C]Martinostat SUVR mediates amyloid-β and tau effects on atrophy and cognitive impairment had the best goodness-of-fit that the other tested models using all model parameters tested. The cells with * show the model with the best goodness-of-fit parameter. For RMSEA, SRMR, AIC, and BIC lower values represent better fit, whereas for CFI a higher value represents a better fit. **Model 1** (meta-model in Fig. 5 and full output in Supplementary Table 1): Model testing the hypothesis that HDAC I reduction mediates the deleterious effect of amyloid-β and tau on brain atrophy and cognitive impairment. **Model 2** (meta-model in Supplementary Fig. 4a and full output in Supplementary Table 2): Model testing the hypothesis that HDAC I reduction is downstream of brain atrophy. **Model 3** (meta-model in Supplementary Fig. 4b and full output in Supplementary Table 3): Model testing the hypothesis that HDAC I reduction precedes amyloid-β and tau pathologies. **Model 4** (meta-model in Supplementary Fig. 4c cand full output in Supplementary Table 4): Model testing the hypothesis that HDAC I reduction succeeds amyloid-β and precedes tau pathology. **Model 5** (meta-model in Supplementary Fig. 5 and full output in Supplementary Table 5): Model testing the classical sequential model of AD progression, without using HDAC I measurements.

### HDAC I in postmortem human brain tissue and animal models of AD

**Fig. 6:**
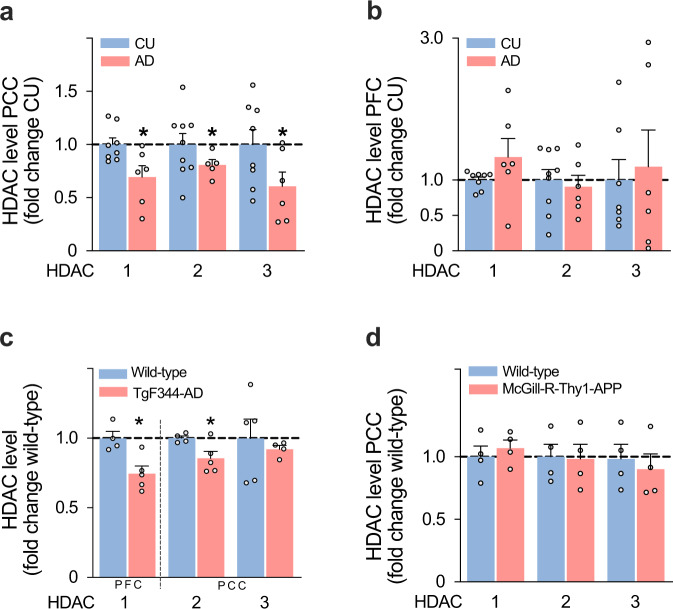
Postmortem analysis shows HDAC I reduction within vulnerable brain regions in Alzheimer’s disease patients and rats overexpressing human amyloid-β and tau pathology. Quantification from brain lysate showed downregulation of HDAC1 (*P* = 0.0364), HDAC2 (*P* = 0.0186), HDAC3 (*P* = 0. 0329) in **a** the posterior cingulate cortex (PCC) of patients with AD (CU, *n* = 9; AD, *n* = 6), whereas no significant difference was found in **b** the prefrontal cortex (PFC) (CU, *n* = 9; AD, *n* = 6) for HDAC1 (*P* = 0.275), HDAC2 (*P* = 0.5885), HDAC3 (*P* = 0. 7346). **c** TgF344-AD rats (*n* = 5 rats each group), which express human amyloid-β and tau pathology, showed a significant reduction in HDAC1 (*P* = 0. 0093), HDAC2 (*P* = 0.0446), and a trend in HDAC3 (*P* = 0. 0613). **d** McGill-R-Thy1-APP rats (*n* = 4 rats each group), which express human amyloid-β without developing tau pathology, showed normal HDAC1 (*P* = 0. 3581), HDAC2 (*P* = 0.8972), and HDAC3 (*P* = 0. 4781) levels. *Indicates *P* < 0.05 (two-sided), one-sample *t*-test; values are mean ± standard error of the mean. HDAC1–3 levels were normalized to beta-actin as a loading control. Uncropped and unprocessed images of the Western Blots are provided as a Source Data File.

We compared the abundance of the HDAC I isoforms 1–3 in postmortem brain tissue from 6 AD (mean age = 76 (SD = 7), 3 males) and 9 CU (mean age = 74 (SD = 9), 4 males) individuals in brain regions with and without HDAC I reduction in AD based on in vivo PET results. Consistent with PET imaging, we found that HDAC1–3 were reduced in the posterior cingulate cortex of patients with AD, whereas no significant reduction was found in the prefrontal cortex (Fig. 6a, b). To examine whether HDACs I reduction is associated with the amyloid-β plus downstream tau pathology, we measured HDACs I in a transgenic rat model that develops only human amyloid-β pathology and in a transgenic rat model that develops both human amyloid-β and downstream tau pathology. We found that the amyloid-β plus tau rat model had a significant reduction in HDAC1 and HDAC2 (Fig. 6c), whereas the single amyloid-β rat model had normal HDACs I level (Fig. 6d).

## Discussion

This study showed that [^11^C]Martinostat HDAC I reduction was highly associated with elevated amyloid-β and tau in AD, which contrasts with previous studies using experimental disease models that predicted HDAC I increase in living patients with AD^2,12,16^. We showed that [^11^C]Martinostat HDAC I reduction predicted longitudinal neurodegeneration and cognitive decline. The HDAC reduction observed in vivo with [^11^C]Martinostat SUVR was supported by postmortem brain tissue from patients with AD and a rat model of amyloid-β plus tau pathology. Together, our results suggest HDAC I reduction as an important epigenetic element associated with AD pathophysiology.

HDAC I reduction in living patients with AD, as described here, challenges previous studies predicting HDAC I upregulation in AD^7,10,17^. Using whole-brain imaging, we showed that HDAC I reduction in our population occurred in brain regions with high amyloid-β and tau concentrations in the precuneus, posterior cingulate, inferior parietal, and lateral temporal cortices, which were not brain regions particularly assessed by previous HDAC I studies. Previous postmortem observations have shown increased HDAC I level in AD^2^. However, these studies used small sample sizes to assesses selected brain areas confined to the frontal and medial temporal cortices^2,13^, which are not regions often affected by high levels of amyloid-β and tau in early AD^25^. While the medial temporal cortex may present low concentrations of amyloid-β, the prefrontal cortex may show low levels of tau pathology in some patients^25^. Therefore, these previous studies may have selected brain regions in which HDAC I levels were not fully affected by AD pathophysiology in their populations. On the other hand, two autopsy studies, also using frontal and temporal regions, showed a clear reduction in HDAC I in AD^22,23^, suggesting a possible susceptibility of HDAC postmortem results to small anatomical variations inside the same region, which supports the importance of using techniques able to assess epigenetic profile across the whole brain. Together, these results suggest that assessing the entire complexity of the epigenetic modification across the whole brain may be crucial for determining the epigenetic profile of patients with complex brain disorders such as AD.

HDACs I reduction in human brain regions with high amyloid-β and tau loads as well as in a rat model presenting human amyloid-β and downstream tau pathology support HDAC I reduction as a possible epigenetic signature associated with the presence of amyloid-β plus downstream tau pathology^26^. Interestingly, normal HDAC I levels in our rat model expressing amyloid-β but not downstream tau pathology, further supported that HDAC I reduction occurs downstream to underlying tau pathology. Although it is well-established that amyloid-β and tau play a central role in AD^1^, HDAC I reduction sheds light on the mechanism by which these pathologies interact in the brain tissue to determine disease progression, which is an important unanswered question in AD.

The strong association between HDAC I reduction and cognitive impairment advocate for a harmful effect of HDAC I reduction in living patients with AD. Supporting these findings, a study showed that p25/Cdk5, a kinase complex involved in AD, inhibits HDAC1 leading to neuronal cell death^27^. Also, HDAC1–3 levels have shown to play an important role in maintaining neuronal plasticity and function^28–30^. Moreover, recent studies suggest that HDAC inhibition leads to pathological tau aggregation^31^, and highlight the benefits of HDAC1 activation in AD^32^. Thus, contradicting the predictions of the mainstream notion^7,10,17^, our results support raise concern that HDAC I inhibitors might result in harm rather than benefit if administered in patients with AD. Indeed, the HDAC I inhibitor valproate has already been associated with faster brain atrophy and cognitive decline in patients with AD^33^.

The strengths of this study include the fact that results were generated using a molecular imaging technology that allows epigenetic quantification in living patients across the entire brain. We supported [^11^C]Martinostat SUVR results using postmortem mRNA expression and immunohistochemistry data. In addition, we replicated our main findings in independent samples analyzed by different groups blind to each other results. One limitation of this study was the lack of a rat model with single tau pathology. Other limitations include the lack of amyloid-β and tau PET for the human participants at the MGH site, which prevented the assessment of AD pathophysiology in this population. As autopsy studies suggest that about a third of patients with a clinical diagnosis of AD dementia do not have AD pathophysiology^34^, it is possible that a portion of the patients with AD studied at MGH did not have underlying AD. Future studies should be designed to elucidate the presence of HDAC I abnormalities in predementia individuals with AD pathophysiology. The individuals studied were motivated to participate in a dementia study. Therefore, these individuals may not represent the general population. Replication studies using larger population-based samples with a longitudinal design and a broader range of ages are needed to confirm our results.

To conclude, our findings indicate HDAC I reduction as an element associated with AD pathophysiology. The fact that HDAC I is associated with DNA repair and neuronal plasticity brings hope that the combination of an HDAC I agonist with the anti-protein aggregate therapies has the potential to rectify underlying epigenetic dysregulations and mitigate AD progression^27–29^.

## Methods

This study was approved by the appropriate institutional ethics committees at the Douglas Mental Health University Institute Research Ethics Board, Montreal Neurological Instituted PET working committee, Partners HealthCare Institutional Review Board, and Massachusetts General Hospital Radioactive Drug Research Committee, and written informed consent was obtained from the participants. All rat work followed the National Institutes of Health guidelines and was approved by the McGill Animal Care Ethics Committee.

### Human participants

We studied 94 participants aged 18–91 years from the community or outpatients at the McGill University Research Centre for Studies in Aging (MCSA, *n* = 48) and Massachusetts General Hospital (MGH, *n* = 46). Participants received financial compensation for their time. To ensure external validity, both sites conducted concomitant but entirely independent studies on design and analysis. At the end of the studies, the two sites presented their results blind to each other’s findings, and these results are the ones presented here. Participants had detailed clinical and cognitive assessments, including the mini-mental state examination (MMSE) and clinical dementia rating (CDR). CU individuals had no objective cognitive impairment and a CDR score of 0. Mild cognitive impairment (MCI) individuals had subjective and objective cognitive impairments, essentially preserved activities of daily living, and a CDR score of 0.5. Mild-to-moderate AD dementia patients had a CDR score between 0.5 and 2, met the National Institute on Aging and the Alzheimer’s Association criteria for probable AD determined by a licensed physician/nurse practitioner, and had amyloid-β and tau abnormalities at the MCSA site. Participants were excluded if they had active substance abuse or inadequately treated conditions, recent head trauma or major surgery, or if they presented any magnetic resonance imaging (MRI)/positron emission tomography (PET) safety contraindication. Patients with AD did not have to discontinue any medication for this study.

### Human material

We obtained from the Douglas-Bell Canada Brain Bank, with the approval of the Brain Bank’s and Douglas Institute’s ethics boards, frozen brain tissues from the posterior cingulate and prefrontal cortices of six patients (3 males, mean age = 76 (7)) with antemortem and postmortem diagnosis of sporadic AD (Consortium to Establish a Registry for Alzheimer’s Disease (CERAD) positive^35^) and nine age-matched CU (4 males, mean age = 74 (9)) (CERAD negative). We also obtained through the Douglas-Bell Canada Brain Bank the brain of a 63-year-old participant with antemortem and postmortem (CERAD positive) diagnosis of sporadic AD who died 22 months after a [^11^C]Martinostat scan. Participants demographic characteristics and diagnosis were obtained from the Brain Bank. The postmortem delay ranged from 8.5 to 21.25 h. Appropriate informed consents were obtained from all brain donors or their families.

### Animal use

The rats were kept in ventilated cages in pairs with environmentally controlled conditions: 12-h light/dark cycle at 21 °C with access to food and water ad libitum. Four transgenic McGill-R-Thy1-APP rats (14–16 months old) with the amyloid-β protein precursor Swedish double (K670N, M671L) and Indiana (V717F) mutations and their respective control littermates were used in this study^36^. McGill-R-Thy1-APP rats present human amyloid-β pathology and cognitive impairment at as early as 6 months, and do not develop tau pathology during their lifetime^36^. Five transgenic TgF344-AD rats (10–12 months old) with the presenilin 1 (PS1ΔE9) and amyloid-β protein precursor Swedish mutations and their respective control littermates were also assessed^37^. TgF344-AD rats manifest human amyloid-β pathology, downstream tau pathology, and cognitive impairment at as early as 6 months^37^. All the groups were matched for sex. Rats were sacrificed by decapitation while anesthetized; the brains were rapidly removed, frozen in liquid nitrogen-isopentanol solution, and stored at −80 °C. The prefrontal and posterior portion of the cingulate (retrosplenial) cortices were dissected and used in the analysis.

### Image methods

At the MCSA site, participants had 3 T MRI (Siemens) and [^11^C]Martinostat^24^, [^18^F]MK-6240^38^, and [^18^F]AZD4694^39^ PET scans acquired with a Siemens high-resolution research tomograph. At the MGH site, MR and [^11^C]Martinostat images were simultaneously acquired on a 3 T Siemens TIM Trio containing a BrainPET insert. [^11^C]Martinostat SUVR for quantifying HDACs I (isoform 1–3) was measured using the telencephalon white matter as reference from 60 to 90 min after tracer injection^24^. [^18^F]MK-6240 SUVR for quantifying tau neurofibrillary tangles and [^18^F]AZD4694 SUVR for quantifying amyloid-β used the cerebellum gray matter as reference and were calculated at 90–110 and 40–70 min after tracer injection, respectively^38,39^. PET images were spatially smoothed to achieve a final 8-mm full width at half maximum resolution. Although both sites used brain-dedicated PET scanners with a high spatial resolution, which makes the images less susceptible to volume effects, we used only partial volume corrected data. Partial volume correction of PET images was performed using the region-based voxel-wise method^40^. Regions-of-interest were tailored using the MNI ICBM atlas. Brain atrophy was assessed with the analysis of gray matter density on T1-weighted images using voxel-based morphometry technique. Hippocampal volume was measured with Freesurfer version 6.0 using the Desikan–Killiany–Tourville gray matter parcellation in a structural T1-weight MRI^41^. For the subset of participants who had full dynamic [^11^C]Martinostat acquisitions from 0 to 90 min after intravenous tracer injection (*n* = 48, CU young = 9, CU elderly = 15, MCI = 8, and AD = 16), we generated R1 relative tracer delivery parametric maps. For the R1 analysis, the dynamic PET data were binned with gradually increasing intervals into 26 frames (6 × 10 s, 6 × 20 s, 2 × 30 s, 1 × 1 min, 5 × 5 min, 6 × 10 min) and reconstructed in units of Becquerel per milliliter. The dynamic data were smoothed with a Gaussian kernel at 8 mm full width at half maximum. To remove extracerebral noise potentially resulting from the volumetric smoothing, we masked the PET data using a probabilistic MNI brain mask with a threshold of 50%. Then, the 4D dynamic data were fitted using the simplified reference tissue model (SRTM)^42^ with the telencephalon white matter as reference to obtain R1 maps—the ratio of K1 in target and reference tissue—which have been found to serve as a reliable surrogate of brain perfusion^43–46^. Based on the kinetic characteristics of [^11^C]Martinostat^24,47^, we assumed that [^11^C]Martinostat-R1 maps offer a proxy of brain perfusion. Also, 13 regions-of-interest were defined according to the Automated Anatomical Labeling human brain atlas as distributed in PMOD to generated regional time–activity curves. Parametric images were validated against the results of averaged time–activity curves to ensure that idiosyncrasies associated with the voxel-wise approach did not influence the results. R1 parametric images were generated using PMOD version 3.3. HDAC gene expression distribution images were derived from microarray data obtained from the open-source Allen Human Brain Atlas^48^, which is composed of mRNA expression intensity values measured on from six healthy human brains (4 males, mean age = 42.5 (13.4), postmortem delay = 20.6 (7) h). The used mRNA expression maps were derived from a Gaussian process^49^.

### Correlation of in vivo [^11^C]Martinostat pet with postmortem HDAC I

The participant was a 63-year-old APOE ε4 positive female with no familiar history of dementia who received the diagnosis of sporadic early-onset AD at the MCSA site. The patient presented with a gradual onset of anterograde episodic memory loss, visual-spatial impairment, perceptual-motor function impairment, and ideomotor apraxia. At the time of [^11^C]Martinostat PET, the patient was severely impaired (MMSE score of 12, CDR score of 1), amyloid-β [^18^F]AZD4694 PET positive, and tau [^18^F]MK-6240 PET positive. The patient was otherwise healthy. The patient died of pneumonia 22 months after the [^11^C]Martinostat PET scan. The brain was examined, and the patient received the neuropathological diagnosis of AD (CERAD positive). Then, we investigated the association of in vivo [^11^C]Martinostat PET with postmortem HDAC I. Based on a previous study^50^, to ensure accurate positioning of the brain regions sampled for the neuropathology evaluation, we indicated the areas to be assessed on the MRI and postmortem brain tissue. The volumes of interest delineated on the MRI were small to match neuropathological sections. The neuroanatomical regions used in the analysis were pons, midbrain, cerebellum dentate, cerebellum vermis, thalamus, basal ganglia, cingulate cortex, hippocampus, amygdala, middle frontal gyrus, superior temporal cortex, inferior parietal cortex, calcarine cortex, lateral occipital gyrus) based on the recommended brain regions by the National Institute on Aging-Alzheimer’s Association guidelines for the neuropathologic assessment of AD^51^. For the in vivo and in vitro analyses, we assessed [^11^C]Martinostat SUVR concentrations as described in the “Image methods” section and postmortem HDAC1–3 levels using immunohistochemistry, respectively. Immunohistochemistry was performed on 5-micron thick formalin-fixed paraffin-embedded sections using a fully automated Discovery Ultra instrument (Ventana Medical Systems, Roche). Sections were treated using Cell Conditioning Solution 1 (CC1; Ventana Medical Systems, Roche) for 64 min before applying the antibodies: HDAC1 (Abcam, catalog number 19845) dilution 1:200, HDAC2 (Abcam, catalog number 16032) dilution 1:200, and HDAC3 (Abcam, catalog number 32369) dilution 1:100 for one hour, followed by Discovery OmniMap DAB anti-rabbit RUO (Ventana Medical Systems, Roche). Slides were digitalized using AperioScope scanner (Leica). Images were quantified using ImageJ v1.51 by an experimenter blind to the equivalent PET value for each region. We averaged the signal intensity in cell nuclei of 40–60 representative cells per section for each HDAC^2^. To obtain postmortem measurements equivalent to [^11^C]Martinostat uptake (agent selective for HDAC I isoforms 1–3), we averaged the nuclear intensities of three HDACs (HDAC1–3).

### Protein extraction, immunoblot, and immunoshistochemistry

Human and rat brain sections were harvested and lysed in RIPA buffer (150 mM NaCl, 0.1% SDS, 0.5% deoxycholate, 50 mM Tris pH 7.5, 1% NP-40) containing complete protease inhibitor (Roche). Following centrifugation (10,000 rpm, 10 min, 4 °C), the total protein was collected from the upper phase. Cell lysates were subjected to gel electrophoresis on 4–12% precast SDS–polyacrylamide gel (SurePAGE^TM^, Bis–Tris, 10 × 8, 4–12%, 12 wells Genscript) in MOPS buffer. Separated proteins were transferred onto nitrocellulose membranes in a wet transfer tank (Bio-Rad) and then probed with antibodies against HDAC1 (abcam, ab19845), HDAC2 (abcam, ab16032) and HDAC3 (abcam, ab32369) at a 1:1000 dilutions in all cases for one hour, followed by a secondary anti-rabbit IgG antibody at 1:5000 dilution for one hour. Whenever possible (i.e., for HDAC1 and HDAC2) knockout-validated antibodies were used. Beta-actin was used as a reference protein (1:5000) and was followed by a secondary anti-mouse IgG antibody at 1:5000. Blots were scanned and analyzed by ChemiDoc gel imaging system (Bio-Rad). Immunohistochemistry was performed on 5-micron-thick formalin-fixed paraffin-embedded sections using a fully automated Discovery Ultra instrument (Ventana). Sections were treated using Cell Conditioning Solution 1 (Ventana) for 64 min before applying the above-mentioned antibodies: HDAC1 and HDAC2 at 1:200 and HDAC3 at 1:100 dilution for one hour, followed by Discovery OmniMap DAB anti-rabbit RUO (Ventana). Slides were digitalized using AperioScope scanner (Leica) and images quantified with ImageJ v1.51. For each HDAC, 40–60 cells per section were analyzed, and the average signal intensity in cell nuclei was obtained.

### Statistics and reproducibility

Statistical analyses were performed using R Statistical software v3.1.2 and MATLAB software v9.2 with VoxelStats package^52^. Biomarker abnormalities were assessed using analysis of variance with post hoc multiple comparisons as well as two-sided *t*-test, whereas associations between biomarkers were tested with regressions and Spearman and Pearson correlation. Linear regression models were adjusted for age, sex, *APOE ε4* status, years of education (models involving cognition), and two-sided false discovery rate corrected for multiple comparisons at *P* < 0.05 (voxel-wise models). Voxel-wise receiver operating characteristic curve, contrasting CU elderly and AD individuals, provided the area under the curve for a diagnosis of AD. Patients’ *z*-score parametric images were obtained anchored on the normative data of the elderly CU population. We evaluated the effects of HDAC I on AD using structural equation modeling with the R package lavaan. Meta-models were created based on expected and hypothesized connections to test the specific hypothesis demonstrated in each figure, and mediation effects sizes were computed^53^. A bootstrap method repeated 1000 times tested the statistical significance of the model’s chi-square and parameter estimates. The fit of the structural equation models was classified as good if: root mean squared error of approximation < 0.05, comparative fit index > 0.97, and standardized root mean square residual < 0.05^53^. The one sample *t*-test (two-sided) analyses had >80% of power to test the difference between groups at a 5% significance level. The correlations presented in the manuscript were two-sided and had over 90% power at a 5% significance. The power of the parameters of the hypothesized SEM—assessed with the pwrSEM software v0.1.2 (https://yilinandrewang.shinyapps.io/pwrSEM/)—were Tau-HDAC = 86% | Amyloid-HDAC = 70% | HDAC-MMSE > 95% | HDAC-Atrophy > 95% | Amyloid-Cognition = 52% | Tau-Cognition = 90% | Atrophy-Cognition = 14% | Amyloid-Atrophy = 14% | Tau-Atrophy = 67%.

### Reporting summary

Further information on research design is available in the Nature Research Reporting Summary linked to this article.

## Supplementary information

Supplementary Information

Reporting Summary

## Data Availability

All requests for raw and processed data should be sent to the corresponding author and will be promptly reviewed by McGill University and Harvard University to verify if the request is subject to any confidentiality obligations. Anonymized data will be shared upon request from a qualified academic investigator. Data and materials will be shared with no restrictions on the availability of raw or processed data via a material transfer agreement. Data are not publicly available due to information that could compromise the privacy of research participants. Gene-expression raw data is publicly available in the Allen Human Brain Atlas dataset (https://human.brain-map.org/static/download) and processed mRNA expression maps can be downloaded at www.meduniwien.ac.at/neuroimaging/mRNA.html. Source data are provided with this paper.

## Source data

Source Data

## Code availability

The codes used in the manuscript can be found at https://github.com/TharickPascoal/Pascoal-et-al-Nat_Comm_2021 and https://zenodo.org/record/6388101^54^.
