# Author Correction: [^11^C]Martinostat PET analysis reveals reduced HDAC I availability in Alzheimer’s disease

**DOI:** 10.1038/s41467-022-33668-0

**Published:** 2022-10-03

**Authors:** Tharick A. Pascoal, Mira Chamoun, Elad Lax, Hsiao-Ying Wey, Monica Shin, Kok Pin Ng, Min Su Kang, Sulantha Mathotaarachchi, Andrea L. Benedet, Joseph Therriault, Firoza Z. Lussier, Frederick A. Schroeder, Jonathan M. DuBois, Baileigh G. Hightower, Tonya M. Gilbert, Nicole R. Zürcher, Changning Wang, Robert Hopewell, Mallar Chakravarty, Melissa Savard, Emilie Thomas, Sara Mohaddes, Sarah Farzin, Alyssa Salaciak, Stephanie Tullo, A. Claudio Cuello, Jean-Paul Soucy, Gassan Massarweh, Heungsun Hwang, Eliane Kobayashi, Bradley T. Hyman, Bradford C. Dickerson, Marie-Christine Guiot, Moshe Szyf, Serge Gauthier, Jacob M. Hooker, Pedro Rosa-Neto

**Affiliations:** 1 Translational Neuroimaging Laboratory, Department of Neurology and Neurosurgery, Faculty of Medicine, The McGill University Research Centre for Studies in Aging, McGill University, Montreal, QC Canada; 2 Departments of Psychiatry and Neurology, University of Pittsburgh School of Medicine, Pittsburgh, PA USA; 3 Departments of Neurology, University of Pittsburgh School of Medicine, Pittsburgh, PA USA; 4 Montreal Neurological Institute, McGill University, Montreal, QC Canada; 5 Department of Molecular Biology, Ariel University, Ariel, Israel; 6 Department of Pharmacology and Therapeutics, McGill University, Montreal, QC Canada; 7 Neurology Athinoula A. Martinos Center for Biomedical Imaging, Department of Radiology, Massachusetts General Hospital, Harvard Medical School, Charlestown, MA USA; 8 Departments of Biological and Biomedical Engineering and Psychiatry, Douglas Mental Health University Institute, Brain Imaging Centre, Montreal, QC Canada; 9 Department of Psychology, McGill University, Montreal, QC Canada; 10 Department of Neurology, Massachusetts General Hospital, Harvard Medical School, Boston, MA USA

**Keywords:** Diagnostic markers, Alzheimer's disease

**Correction to**: *Nature Communications* 10.1038/s41467-022-30653-5, published online 19 July 2022

In this article the funding from ‘Alzheimer’s Drug Discovery Foundation (ADDF)’ was omitted. The original article has been corrected.

