# Supplementary material for: [11C]Martinostat PET analysis reveals reduced HDAC I availability in Alzheimer’s disease
Source: Nat Commun. 2022 Jul 19;13:4171. doi: 10.1038/s41467-022-30653-5 (PMC9296476; doi:10.1038/s41467-022-30653-5)
Supplement: Supplementary file 1 — Supplementary Information [file 41467_2022_30653_MOESM1_ESM.pdf]

Supplementary Information

[11C]Martinostat PET analysis reveals reduced HDAC I availability in Alzheimer's disease

Tharick A. Pascoal, Mira Chamoun, Elad Lax, Hsiao-Ying Wey, Monica Shin, Kok Pin Ng, Min Su Kang, Sulantha Mathotaarachchi, Andrea L. Benedet, Joseph Therriault, Firoza Z. Lussier, Frederick A. Schroeder, Jonathan M. DuBois, Baileigh G. Hightower, Tonya M. Gilbert, Nicole R. Zürcher, Changning Wang, Robert Hopewell, Mallar Chakravarty, Melissa Savard, Emilie Thomas, Sara Mohaddes, Sarah Farzin, Alyssa Salaciak, Stephanie Tullo, A. Claudio Cuello, Jean-Paul Soucy, Gassan Massarweh, Heungsun Hwang, Eliane Kobayashi, Bradley T. Hyman, Bradford C. Dickerson, Marie-Christine Guiot, Moshe Szyf, Serge Gauthier, Jacob M. Hooker, and Pedro Rosa-Neto.

SUPPLEMENTARY FIGURES

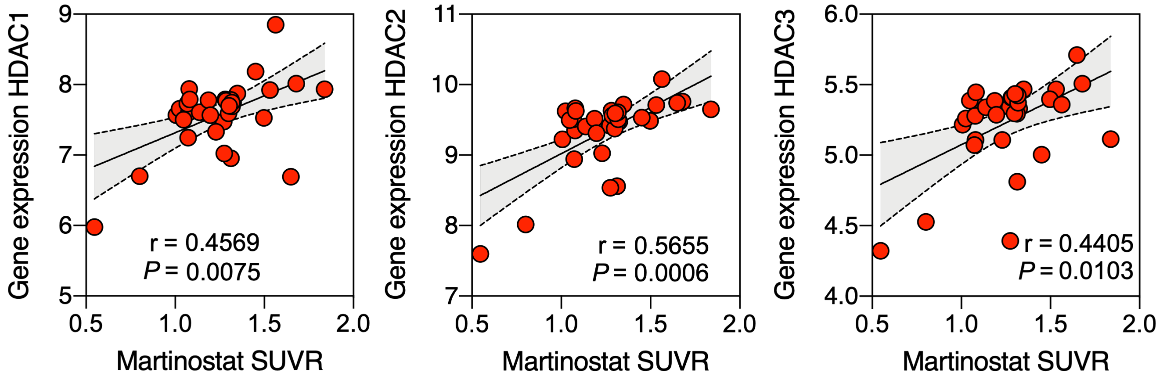

Supplementary Figure 1 | Brain co-localization of in vivo [11C]Martinostat SUVR and HDAC1-3 Allen Gene Expression. Two-sided Spearman's rank correlation between HDAC1-3 mRNA expressions in 6 CU individuals from the Allen Human Brain Atlas and [11C]Martinostat SUVR distribution in the corresponding brain regions. The error bars represent 95% confidence interval. Source data are provided as a Source Data file.

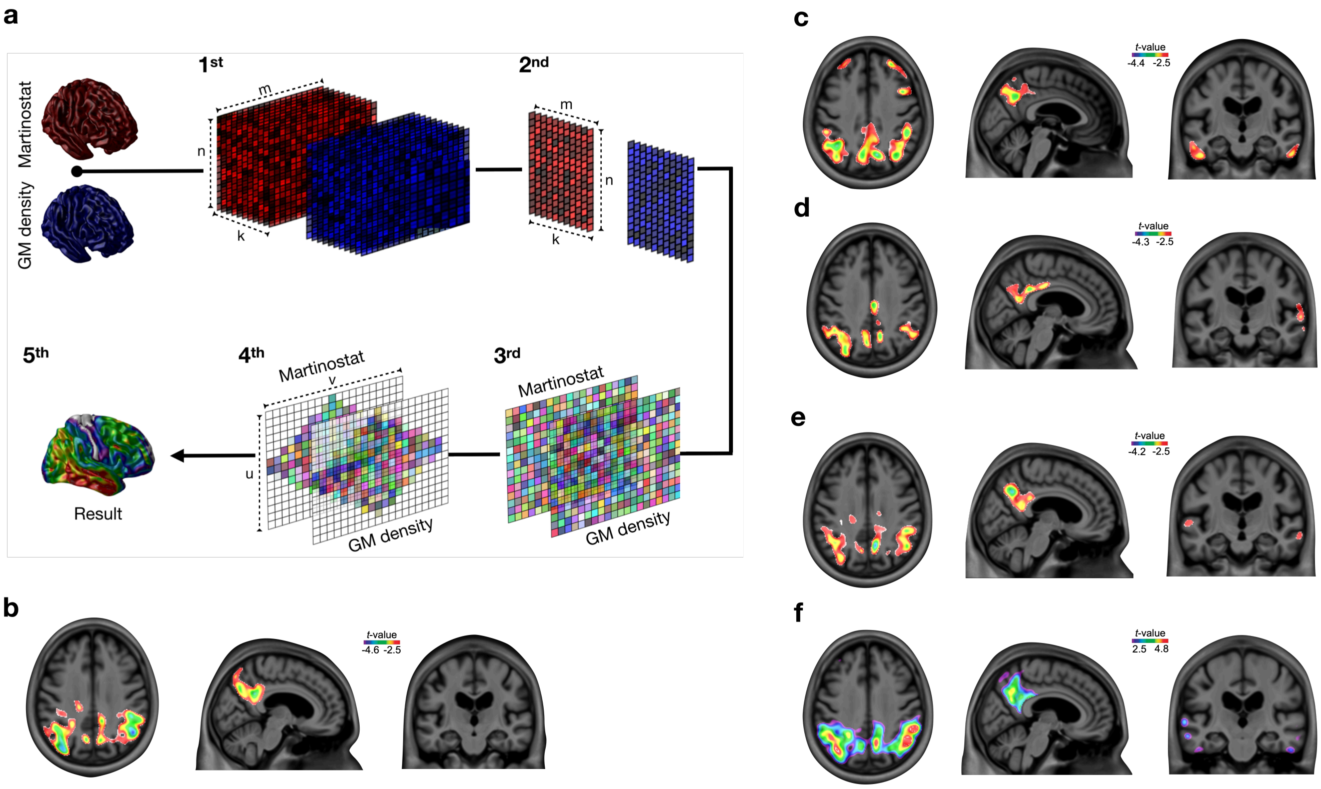

Supplementary Figure 2 | [^{11}C]Martinostat SUVR results remained after correction for atrophy. (**a**) The illustration summarizes the analytical pipeline performed to correct the voxel-wise statistical models presented in the manuscript for atrophy (gray matter density) using MATLAB software version 9.2 with VoxelStats package. Briefly, (1st) the brain images were retrieved from 3D image space to 2D matrices for every subject using artificial parcellation. (2nd) Statistical models were performed at every brain voxel in a 2D space, accounting for the gray matter density values. (3rd) The statistical matrices were generated from the models' results and (4th) transformed back to the 3D image space. Finally, (5th) we generated 3D images displaying models result. k = individuals, u = image slice, v = slice elements, m = artificial parcellation, n = elements in each parcellation. *T*-statistical parametric maps, overlaid on an MRI template, show the regions where [^{11}C]Martinostat uptake corrected for colocalized atrophy was reduced in AD (*n* = 16) compared to CU elderly (*n* = 15) at (**b**) the MCSA site, and reduced in AD (*n* = 10) compared to CU elderly (*n* = 13) at (**c**) the MGH site. Also, regions where [^{11}C]Martinostat uptake was associated with (**d**) amyloid-β PET (*n* = 45), (**e**) tau PET (*n* = 44), and (**f**) cognition measured with MMSE (*n* = 48). The results were two-tailed false discovery rate corrected at $P < 0.05$.

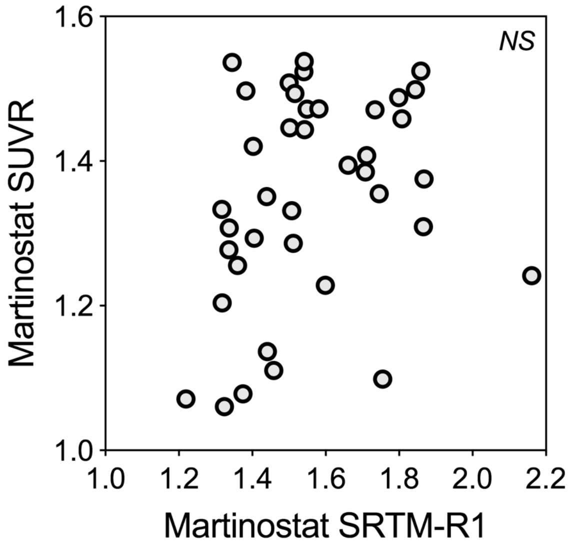

Supplementary Figure 3 | [^{11}C]Martinostat SUVR was not correlated with relative cerebral blood flow. The linear regression shows no significant association (β = 0.19, R^2 = 0.08, *P* = 0.08 (trend)) between [^{11}C]Martinostat SUVR and [^{11}C]Martinostat SRTM-R1 relative tracer delivery values in the brain regions with significant [^{11}C]Martinostat reduction in AD (see Fig. 2c) (among subset of elderly individuals who underwent full dynamic [^{11}C]Martinostat acquisitions; *n* = 39; CU elderly = 15, MCI = 8, and AD = 16). This finding supports that changes in brain perfusion did not drive [^{11}C]Martinostat uptake. Source data are provided as a Source Data file.

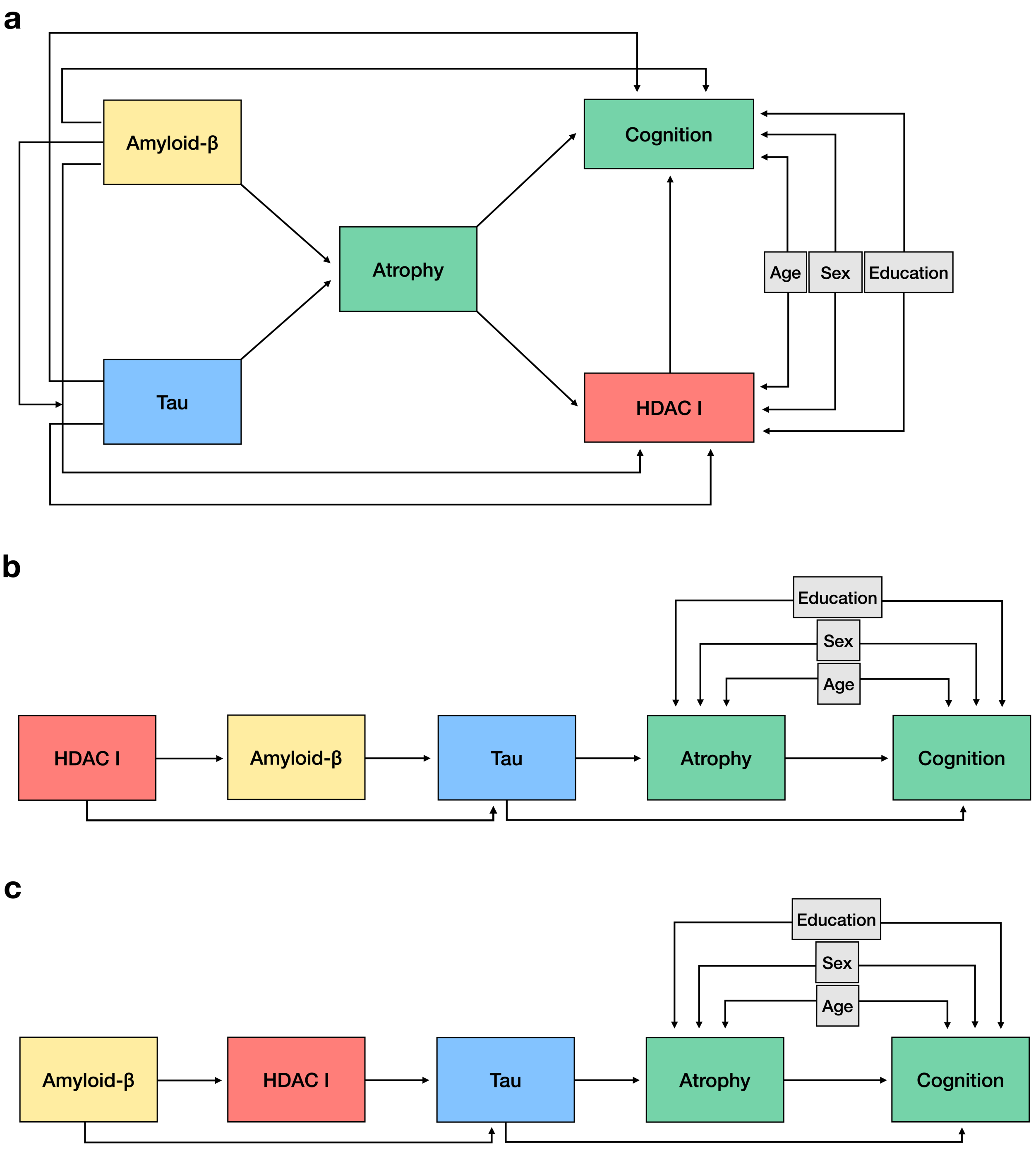

Supplementary Figure 4 | Structural equation models testing HDAC I reduction upstream to amyloid-β and tau or downstream to atrophy fitted the data poorly. (**a**) The model testing whether HDAC I reduction occurs downstream to atrophy fitted the data poorly (n = 44, X^2 = 21.677, degrees of freedom = 6, P = 0.001, root mean squared error of approximation (RMSEA) = 0.244 (90% confidence interval (CI) [0.139-0.358]), standardized root mean square residual (SRMR) = 0.123, comparative fit index (CFI) = 0.935, Akaike information criterion (AIC) = 895.476, and Bayesian information criterion (BIC) = 931.159); complete results of the model are presented in Supplementary Table 2. (**b**) The model testing

whether HDAC I reduction precedes amyloid-β and tau fitted the data poorly (n = 44, X^2 = 76.719, degrees of freedom = 10, P < 0.001, RMSEA = 0.389 (90% CI [0.311-0.473]), SRMR = 0.090, CFI = 0.724, AIC = 942.515, and BIC = 971.062); complete results of the model are presented in Supplementary Table 3. (**c**) The model testing whether HDAC I reduction precedes amyloid-β but succeeds tau fitted the data poorly (n = 44, X^2 = 75.966, degrees of freedom = 10, P < 0.001, RMSEA = 0.387 (90% CI [0.309-0.471]), SRMR = 0.089, CFI = 0.726, AIC = 941.764, and BIC = 970.311); complete results of the model are presented in Supplementary Table 4. The imaging biomarker values were extracted from brain regions with significant [11C]Martinostat reduction in AD. The PET biomarker values used in this model were adjusted for age. Cognition and atrophy were measured with MMSE and gray matter density, respectively.

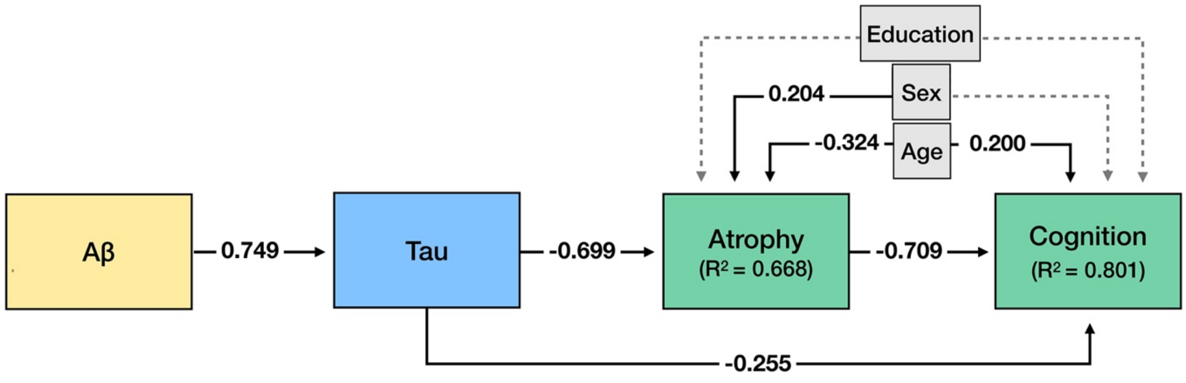

Supplementary Figure 5 | Structural equation model testing the classical sequential model of Alzheimer's disease progression. Solid arrows represent significant effects, whereas dashed lines represent non-significant effects. The effect sizes (β estimates) presented in the figure are standardized and therefore may be compared. The classical sequential model of AD progression fitted the data well (n = 44, X^2 = 5.680, degrees of freedom = 5, P = 0.339, RMSEA = 0.056 (90% CI [0.000-0.223]), SRMR = 0.037, CFI = 0.995, AIC = 998.189, and BIC = 1021.383). The imaging biomarker values were extracted from regions showing significant [11C]Martinostat reduction in AD. The PET biomarker values were adjusted for age. Cognition and atrophy were measured with MMSE and gray matter density, respectively. The complete results of the model are presented in Supplementary Table 5.

SUPPLEMENTARY TABLES

Supplementary Table 1 | Full results of the structural equation model presented in Figure 5.

```
  lavaan (0.5-23.1097) converged normally after 106 iterations

                                                  Used       Total
  Number of observations                            44          48

  Estimator                                         ML
  Minimum Function Test Statistic                1.818
  Degrees of freedom                                 6
  P-value (Chi-square)                           0.936
  P-value (Bollen-Stine Bootstrap)               1.000

  Model test baseline model:
  Minimum Function Test Statistic              266.909
  Degrees of freedom                                22
  P-value                                        0.000

  User model versus baseline model:
  Comparative Fit Index (CFI)                    1.000
  Tucker-Lewis Index (TLI)                       1.063

  Loglikelihood and Information Criteria:
  Loglikelihood user model (H0)               -415.816
  Loglikelihood unrestricted model (H1)       -414.907

  Number of free parameters                         20
  Akaike (AIC)                                 871.632
  Bayesian (BIC)                               907.316
  Sample-size adjusted Bayesian (BIC)          844.643

  Root Mean Square Error of Approximation:
  RMSEA                                          0.000
  90 Percent Confidence Interval          0.000  0.049
  P-value RMSEA <= 0.05                          0.950

  Standardized Root Mean Square Residual:
  SRMR                                           0.029

  Parameter Estimates:
  Information                                 Observed
  Standard Errors                            Bootstrap
  Number of requested bootstrap draws             1000
  Number of successful bootstrap draws            1000

Regressions:      Estimate  Std.Err  z-value  P(>|z|)   Std.lv  Std.all
HDAC ~
  AB        (a)     -0.088    0.038   -2.337    0.019   -0.088   -0.361
  TAU       (b)     -0.058    0.024   -2.419    0.016   -0.058   -0.446
MMSE ~
  HDAC      (c)     32.467    5.805    5.593    0.000   32.467    0.755
  AB        (d)      1.379    0.806    1.711    0.087    1.379    0.132
  TAU       (e)     -1.472    0.537   -2.740    0.006   -1.472   -0.262
  GM        (f)      5.883   12.554    0.469    0.639    5.883    0.069
  Education          0.188    0.079    2.372    0.018    0.188    0.116
  Sex               -0.325    0.961   -0.339    0.735   -0.325   -0.022
  Age               -0.016    0.034   -0.487    0.626   -0.016   -0.029
GM  ~
  HDAC      (g)      0.317    0.051    6.238    0.000    0.317    0.627
  AB        (h)     -0.007    0.013   -0.557    0.578   -0.007   -0.060
  TAU       (i)     -0.014    0.007   -2.166    0.030   -0.014   -0.214
  Education          0.001    0.001    0.876    0.381    0.001    0.061
  Sex                0.028    0.012    2.387    0.017    0.028    0.157
  Age               -0.002    0.000   -4.790    0.000   -0.002   -0.349
TAU ~
  AB                 1.395    0.227    6.143    0.000    1.395    0.749

Variances:         Estimate  Std.Err  z-value  P(>|z|)   Std.lv  Std.all
  .HDAC               0.013    0.003    3.866    0.000    0.013    0.430
  .MMSE               5.018    1.108    4.528    0.000    5.018    0.090
  .GM                 0.001    0.000    4.696    0.000    0.001    0.141
  .TAU                0.776    0.153    5.077    0.000    0.776    0.439

R-Square:        Estimate
  HDAC              0.570
  MMSE              0.910
  GM                0.859
  TAU               0.561

Defined Parameters:
                  Estimate  Std.Err  z-value  P(>|z|)   Std.lv  Std.all
  AB_HC_MMSE        -2.851    1.234   -2.311    0.021   -2.851   -0.273
  TAU_HC_MMSE       -1.890    0.907   -2.084    0.037   -1.890   -0.337
  AB_HC_GM          -0.028    0.013   -2.204    0.028   -0.028   -0.226
  TAU_HC_GM         -0.018    0.009   -2.165    0.030   -0.018   -0.280
  AB_GM_MMSE        -0.043    0.192   -0.226    0.821   -0.043   -0.004
  TAU_GM_MMSE       -0.083    0.225   -0.369    0.712   -0.083   -0.015
  AB_HC_GM_MMSE     -0.164    0.403   -0.407    0.684   -0.164   -0.016
  TAU_HC_GM_MMSE    -0.109    0.259   -0.420    0.675   -0.109   -0.019
  total             -5.186    1.036   -5.004    0.000   -5.186   -1.170
```

Supplementary Table 2 | Full results of the structural equation model presented in Supplementary Figure 4a.

```
lavaan (0.5-23.1097) converged normally after 109 iterations

                                                  Used       Total
  Number of observations                            44          48

  Estimator                                         ML
  Minimum Function Test Statistic               21.677
  Degrees of freedom                                 6
  P-value (Chi-square)                           0.001

Model test baseline model:

  Minimum Function Test Statistic              263.032
  Degrees of freedom                                22
  P-value                                        0.000

User model versus baseline model:

  Comparative Fit Index (CFI)                    0.935
  Tucker-Lewis Index (TLI)                       0.762

Loglikelihood and Information Criteria:

  Loglikelihood user model (H0)               -427.738
  Loglikelihood unrestricted model (H1)       -416.899

  Number of free parameters                         20
  Akaike (AIC)                                 895.476
  Bayesian (BIC)                               931.159
  Sample-size adjusted Bayesian (BIC)          868.487

Root Mean Square Error of Approximation:

  RMSEA                                          0.244
  90 Percent Confidence Interval          0.139  0.358
  P-value RMSEA <= 0.05                          0.003

Standardized Root Mean Square Residual:

  SRMR                                           0.123

Parameter Estimates:

  Information                                 Expected
  Standard Errors                             Standard

Regressions:
                   Estimate  Std.Err  z-value  P(>|z|)   Std.lv  Std.all
  GM ~
    AB        (a)   -0.044    0.018   -2.456    0.014   -0.044   -0.352
    TAU       (b)   -0.030    0.010   -3.130    0.002   -0.030   -0.449
  MMSE ~
    GM        (c)    7.316    9.606    0.762    0.446    7.316    0.082
    AB        (d)    1.003    0.742    1.352    0.176    1.003    0.090
    TAU       (e)   -1.297    0.406   -3.196    0.001   -1.297   -0.218
    HDAC      (f)   31.793    4.456    7.135    0.000   31.793    0.778
    Education        0.171    0.079    2.162    0.031    0.171    0.099
    Sex             -0.331    0.727   -0.456    0.649   -0.331   -0.021
    Age             -0.013    0.032   -0.401    0.688   -0.013   -0.022
  HDAC ~
    GM        (g)    1.718    0.196    8.748    0.000    1.718    0.783
    AB        (h)   -0.016    0.025   -0.622    0.534   -0.016   -0.057
    TAU       (i)   -0.007    0.014   -0.542    0.588   -0.007   -0.051
    Education       -0.001    0.003   -0.334    0.738   -0.001   -0.021
    Sex             -0.034    0.024   -1.418    0.156   -0.034   -0.088
    Age              0.004    0.001    4.651    0.000    0.004    0.284
  TAU ~
    AB               1.332    0.200    6.672    0.000    1.332    0.709

Variances:
                   Estimate  Std.Err  z-value  P(>|z|)   Std.lv  Std.all
   .GM               0.003    0.001    4.690    0.000    0.003    0.450
   .MMSE             5.171    1.103    4.690    0.000    5.171    0.083
   .HDAC             0.006    0.001    4.690    0.000    0.006    0.159
   .TAU              0.878    0.187    4.690    0.000    0.878    0.497

Defined Parameters:
                   Estimate  Std.Err  z-value  P(>|z|)   Std.lv  Std.all
    AB_GM_MMSE      -0.321    0.441   -0.727    0.467   -0.321   -0.029
    TAU_GM_MMSE     -0.218    0.294   -0.740    0.459   -0.218   -0.037
    AB_GM_HC        -0.075    0.032   -2.364    0.018   -0.075   -0.276
    TAU_GM_HC       -0.051    0.017   -2.947    0.003   -0.051   -0.352
    AB_HC_MMSE      -0.494    0.798   -0.620    0.535   -0.494   -0.044
    TAU_HC_MMSE     -0.236    0.436   -0.540    0.589   -0.236   -0.040
    AB_GM_HC_MMSE   -2.394    1.067   -2.244    0.025   -2.394   -0.215
    TAU_GM_HC_MMSE  -1.624    0.596   -2.724    0.006   -1.624   -0.273
    total           -5.413    1.083   -4.996    0.000   -5.413   -1.265
```

Supplementary Table 3 | Full results of the structural equation model presented in Supplementary Figure 4b.

```
lavaan (0.5-23.1097) converged normally after 80 iterations

                                                  Used       Total
  Number of observations                            44          48

  Estimator                                         ML
  Minimum Function Test Statistic               76.717
  Degrees of freedom                                10
  P-value (Chi-square)                           0.000

Model test baseline model:

  Minimum Function Test Statistic              263.784
  Degrees of freedom                                22
  P-value                                        0.000

User model versus baseline model:

  Comparative Fit Index (CFI)                    0.724
  Tucker-Lewis Index (TLI)                       0.393

Loglikelihood and Information Criteria:

  Loglikelihood user model (H0)               -455.258
  Loglikelihood unrestricted model (H1)       -416.899

  Number of free parameters                         16
  Akaike (AIC)                                 942.515
  Bayesian (BIC)                               971.062
  Sample-size adjusted Bayesian (BIC)          920.925

Root Mean Square Error of Approximation:

  RMSEA                                          0.389
  90 Percent Confidence Interval          0.311  0.473
  P-value RMSEA <= 0.05                          0.000

Standardized Root Mean Square Residual:

  SRMR                                           0.090

Parameter Estimates:

  Information                                 Expected
  Standard Errors                             Standard

Regressions:
                   Estimate  Std.Err  z-value  P(>|z|)   Std.lv  Std.all
  GM  ~
    TAU       (a)    -0.047    0.006   -7.998    0.000   -0.047   -0.697
    Education         0.003    0.002    1.466    0.143    0.003    0.133
    Sex               0.036    0.016    2.274    0.023    0.036    0.203
    Age              -0.002    0.001   -3.671    0.000   -0.002   -0.323
  MMSE ~
    GM        (b)    59.863    9.861    6.071    0.000   59.863    0.715
    TAU       (c)    -1.440    0.598   -2.409    0.016   -1.440   -0.257
    Education         0.138    0.118    1.167    0.243    0.138    0.085
    Sex              -1.331    1.105   -1.205    0.228   -1.331   -0.089
    Age               0.113    0.044    2.562    0.010    0.113    0.201
  TAU ~
    AB        (d)     0.767    0.250    3.065    0.002    0.767    0.408
    HDAC      (e)    -3.307    1.020   -3.241    0.001   -3.307   -0.432
  AB ~
    HDAC             -2.842    0.441   -6.450    0.000   -2.842   -0.697

Variances:
                   Estimate  Std.Err  z-value  P(>|z|)   Std.lv  Std.all
   .GM                0.003    0.001    4.690    0.000    0.003    0.331
   .MMSE             11.205    2.389    4.690    0.000   11.205    0.202
   .TAU               0.709    0.151    4.690    0.000    0.709    0.401
   .AB                0.257    0.055    4.690    0.000    0.257    0.514

Defined Parameters:
                   Estimate  Std.Err  z-value  P(>|z|)   Std.lv  Std.all
    TAU_GM_MMSE      -2.793    0.578   -4.835    0.000   -2.793   -0.498
    total            -2.793    0.578   -4.835    0.000   -2.793   -0.498
```

Supplementary Table 4 | Full results of the structural equation model presented in Supplementary Figure 4c.

```
lavaan (0.5-23.1097) converged normally after 90 iterations

                                                  Used       Total
  Number of observations                            44          48

  Estimator                                         ML
  Minimum Function Test Statistic               75.966
  Degrees of freedom                                10
  P-value (Chi-square)                           0.000

Model test baseline model:

  Minimum Function Test Statistic              263.032
  Degrees of freedom                                22
  P-value                                        0.000

User model versus baseline model:

  Comparative Fit Index (CFI)                    0.726
  Tucker-Lewis Index (TLI)                       0.398

Loglikelihood and Information Criteria:

  Loglikelihood user model (H0)               -454.882
  Loglikelihood unrestricted model (H1)       -416.899

  Number of free parameters                         16
  Akaike (AIC)                                 941.764
  Bayesian (BIC)                               970.311
  Sample-size adjusted Bayesian (BIC)          920.174

Root Mean Square Error of Approximation:

  RMSEA                                          0.387
  90 Percent Confidence Interval          0.309  0.471
  P-value RMSEA <= 0.05                          0.000

Standardized Root Mean Square Residual:

  SRMR                                           0.089

Parameter Estimates:

  Information                                 Expected
  Standard Errors                             Standard

Regressions:
                   Estimate  Std.Err  z-value  P(>|z|)   Std.lv  Std.all
  GM  ~
    TAU        (a)   -0.047    0.006   -7.965    0.000   -0.047   -0.701
    Education         0.003    0.002    1.463    0.143    0.003    0.134
    Sex               0.036    0.016    2.274    0.023    0.036    0.205
    Age              -0.002    0.001   -3.671    0.000   -0.002   -0.325
  MMSE ~
    GM         (b)   59.863    9.861    6.071    0.000   59.863    0.709
    TAU        (c)   -1.440    0.599   -2.405    0.016   -1.440   -0.257
    Education         0.138    0.119    1.165    0.244    0.138    0.085
    Sex              -1.331    1.105   -1.204    0.228   -1.331   -0.089
    Age               0.113    0.044    2.562    0.010    0.113    0.201
  TAU ~
    AB         (d)    0.767    0.250    3.065    0.002    0.767    0.408
    HDAC       (e)   -3.307    1.020   -3.241    0.001   -3.307   -0.432
  HDAC ~
    AB               -0.171    0.027   -6.450    0.000   -0.171   -0.697

Variances:
                   Estimate  Std.Err  z-value  P(>|z|)   Std.lv  Std.all
   .GM                0.003    0.001    4.690    0.000    0.003    0.335
   .MMSE             11.205    2.389    4.690    0.000   11.205    0.201
   .TAU               0.709    0.151    4.690    0.000    0.709    0.401
   .HDAC              0.015    0.003    4.690    0.000    0.015    0.514

Defined Parameters:
                   Estimate  Std.Err  z-value  P(>|z|)   Std.lv  Std.all
    TAU_GM_MMSE      -2.793    0.578   -4.828    0.000   -2.793   -0.498
    total            -2.793    0.578   -4.828    0.000   -2.793   -0.498
```

Supplementary Table 5 | Full results of the structural equation model presented in Supplementary Figure 5.

```
  lavaan (0.5-23.1097) converged normally after 74 iterations

  Used       Total
  Number of observations                            44         48

  Estimator                                         ML
  Minimum Function Test Statistic                5.680
  Degrees of freedom                                 5
  P-value (Chi-square)                           0.339

  Model test baseline model:

  Minimum Function Test Statistic              159.470
  Degrees of freedom                                15
  P-value                                        0.000

  User model versus baseline model:

  Comparative Fit Index (CFI)                    0.995
  Tucker-Lewis Index (TLI)                       0.986

  Loglikelihood and Information Criteria:

  Loglikelihood user model (H0)               -486.095
  Loglikelihood unrestricted model (H1)       -483.255

  Number of free parameters                         13
  Akaike (AIC)                                 998.189
  Bayesian (BIC)                              1021.383
  Sample-size adjusted Bayesian (BIC)          980.647

  Root Mean Square Error of Approximation:

  RMSEA                                          0.056
  90 Percent Confidence Interval          0.000  0.223
  P-value RMSEA <= 0.05                          0.403

  Standardized Root Mean Square Residual:

  SRMR                                           0.037

  Parameter Estimates:

  Information                                 Expected
  Standard Errors                             Standard

Regressions:      Estimate  Std.Err  z-value  P(>|z|)   Std.lv  Std.all
TAU ~
  AB        (a)    1.395    0.186    7.492    0.000    1.395    0.749
GM  ~
  TAU       (b)   -0.047    0.006   -7.914    0.000   -0.047   -0.699
  Education        0.003    0.002    1.456    0.145    0.003    0.133
  Sex              0.036    0.016    2.272    0.023    0.036    0.204
  Age             -0.002    0.001   -3.671    0.000   -0.002   -0.324
MMSE ~
  GM        (c)   59.863    9.861    6.071    0.000   59.863    0.709
  TAU             -1.440    0.600   -2.399    0.016   -1.440   -0.255
  Education        0.138    0.119    1.160    0.246    0.138    0.084
  Sex             -1.331    1.106   -1.204    0.229   -1.331   -0.088
  Age              0.113    0.044    2.562    0.010    0.113    0.200

Variances:       Estimate  Std.Err  z-value  P(>|z|)   Std.lv  Std.all
  TAU              0.776    0.166    4.690    0.000    0.776    0.439
  GM               0.003    0.001    4.690    0.000    0.003    0.332
  MMSE            11.205    2.389    4.690    0.000   11.205    0.199

R-Square:        Estimate
  TAU              0.561
  GM               0.668
  MMSE             0.801

Defined Parameters:
                  Estimate  Std.Err  z-value  P(>|z|)   Std.lv  Std.all
  AB_GM_MMSE      -3.895    0.961   -4.052    0.000   -3.895   -0.371
  total           -3.895    0.961   -4.052    0.000   -3.895   -0.371
```
