# Supplementary material for: [11C]Martinostat PET analysis reveals reduced HDAC I availability in Alzheimer’s disease
Source: Nat Commun. 2022 Jul 19;13:4171. doi: 10.1038/s41467-022-30653-5 (PMC9296476; doi:10.1038/s41467-022-30653-5)
Supplement: Supplementary file 2 — Reporting Summary [file 41467_2022_30653_MOESM2_ESM.pdf]

nature portfolio

Corresponding author(s): Pedro Rosa-Neto
Last updated by author(s): May 3, 2022

Reporting Summary

Nature Portfolio wishes to improve the reproducibility of the work that we publish. This form provides structure for consistency and transparency in reporting. For further information on Nature Portfolio policies, see our Editorial Policies and the Editorial Policy Checklist.

Statistics

For all statistical analyses, confirm that the following items are present in the figure legend, table legend, main text, or Methods section.

n/a	Confirmed	
☐	☒	The exact sample size (*n*) for each experimental group/condition, given as a discrete number and unit of measurement
☐	☒	A statement on whether measurements were taken from distinct samples or whether the same sample was measured repeatedly
☐	☒	The statistical test(s) used AND whether they are one- or two-sided<br>*Only common tests should be described solely by name; describe more complex techniques in the Methods section.*
☐	☒	A description of all covariates tested
☐	☒	A description of any assumptions or corrections, such as tests of normality and adjustment for multiple comparisons
☐	☒	A full description of the statistical parameters including central tendency (e.g. means) or other basic estimates (e.g. regression coefficient) AND variation (e.g. standard deviation) or associated estimates of uncertainty (e.g. confidence intervals)
☐	☒	For null hypothesis testing, the test statistic (e.g. *F*, *t*, *r*) with confidence intervals, effect sizes, degrees of freedom and *P* value noted<br>*Give P values as exact values whenever suitable.*
☒	☐	For Bayesian analysis, information on the choice of priors and Markov chain Monte Carlo settings
☐	☒	For hierarchical and complex designs, identification of the appropriate level for tests and full reporting of outcomes
☐	☒	Estimates of effect sizes (e.g. Cohen's *d*, Pearson's *r*), indicating how they were calculated

Our web collection on statistics for biologists contains articles on many of the points above.

Software and code

Policy information about availability of computer code

Data collection	ChemiDoc gel imaging system (Bio-Rad, Mississauga, ON).
Data analysis	R Statistical Software Package version 3.1.2; Matlab version 9.2 with VoxelStats package (https://github.com/sulantha2006/VoxelStats); Medical Image NetCDF software toolbox version 2.2.0 (www.bic.mni.mcgill.ca/ServicesSoftware/MINC); pwrSEM software v0.1.2 https://yilinandrewang.shinyapps.io/pwrSEM/; ImageJ v1.51; Freesurfer version 6.0.

For manuscripts utilizing custom algorithms or software that are central to the research but not yet described in published literature, software must be made available to editors and reviewers. We strongly encourage code deposition in a community repository (e.g. GitHub). See the Nature Portfolio guidelines for submitting code & software for further information.

Data

Policy information about availability of data

All manuscripts must include a data availability statement. This statement should provide the following information, where applicable:
- Accession codes, unique identifiers, or web links for publicly available datasets
- A description of any restrictions on data availability
- For clinical datasets or third party data, please ensure that the statement adheres to our policy

All requests for raw and processed data should be sent to the corresponding author and will be promptly reviewed by McGill University and Harvard University to verify if the request is subject to any confidentiality obligations. Anonymized data will be shared upon request from a qualified academic investigator. Data and materials will be shared with no restrictions on the availability of raw or processed data via a material transfer agreement. Data are not publicly available due to information that could compromise the privacy of research participants. Gene-expression raw data is publicly available in the Allen Human Brain Atlas dataset (https://human.brain-map.org/static/download) and processed mRNA expression maps can be downloaded at www.meduniwien.ac.at/neuroimaging/mRNA.html.

Source data are provided with this paper.

Field-specific reporting

Please select the one below that is the best fit for your research. If you are not sure, read the appropriate sections before making your selection.

☒ Life sciences ☐ Behavioural & social sciences ☐ Ecological, evolutionary & environmental sciences

For a reference copy of the document with all sections, see nature.com/documents/nr-reporting-summary-flat.pdf

Life sciences study design

All studies must disclose on these points even when the disclosure is negative.

Sample size	Based on the literature showing class I HDAC increase in AD compared to matched CN, we estimated a Cohen's d of 1.2 to differentiate these two groups. Thus, we assumed that a study with 80% of power at a 5% significance level would require as little as 12 individuals per arm to test a difference between AD and matched CN using Martinostat (two-tailed t-test). The one sample t-test (two-tailed) analyses had > 80% of power to test the difference between groups at a 5% significance level. Correlations presented in the manuscript were two-sided and had over 90% power at a 5% significance level. The power analyses for the SEM mediations were perform using the pwrSEM software v0.1.2 (https://yilinandrewang.shinyapps.io/pwrSEM/), and the power for the parameters of the hypothesized model were Tau-HDAC = 86% | Amyloid-HDAC = 70% | HDAC-MMSE > 95% | HDAC-Atrophy > 95% | Amyloid-Cognition = 52% | Tau-Cognition = 90% | Atrophy-Cognition = 14% | Amyloid-Atrophy = 14% | Tau-Atrophy = 67%.
Data exclusions	There were no identified outliers in the in vivo data. To reduce noise, we predetermined exclusion of outliers following the robust formal test of Iglewicz and Hoaglin for multiple outliers for the western blot analysis (double-sided test with |z> = 3.5|, recommended). This was used for all western blot experiments and exclusions were defined before any analysis between groups. Exclusions are described in the sample size in each analysis in Fig. 4. Humans: 1 CN (HDAC1 and 3 (2 PFC)); 1 AD HDAC2 | TgF344-AD: 1 WT HDAC1 and 2; 1 Tg HDAC3 | McGill-R-Thy1-APP: 1 WT HDAC2.
Replication	In order to ensure external validity, sites conducted concomitant but entirely independent studies on design and analysis. At the end of both studies, the two sites presented their final results blind to each other's findings and these results are the ones presented in this study. All the attempts of replication were successful.
Randomization	Allocation on groups was based on diagnosis (humans) and presence or absence of mutation (animal models), so no randomization was performed.
Blinding	All the experimenters were performed entirely blind to groups and other results.

Reporting for specific materials, systems and methods

We require information from authors about some types of materials, experimental systems and methods used in many studies. Here, indicate whether each material, system or method listed is relevant to your study. If you are not sure if a list item applies to your research, read the appropriate section before selecting a response.

Materials & experimental systems

n/a	Involved in the study	
☐	☒	Antibodies
☒	☐	Eukaryotic cell lines
☒	☐	Palaeontology and archaeology
☐	☒	Animals and other organisms
☐	☒	Human research participants
☒	☐	Clinical data
☒	☐	Dual use research of concern

Methods

n/a	Involved in the study	
☒	☐	ChIP-seq
☒	☐	Flow cytometry
☒	☐	MRI-based neuroimaging

Antibodies

Antibodies used	HDAC1 (supplier: ABCAM; cat. number: ab19845; clone name: Rabbit Polyclonal, LOT number: GR3194344-2) | HDAC2 (supplier: ABCAM; cat. number: ab16032; clone name: Rabbit Polyclonal; LOT number: GR3196806-1)) | HDAC3 (supplier: ABCAM; cat. number: [Y415] ab32369; clone name: Rabbit Monoclonal to HDAC3, LOT: GR320398-9) | Secondary anti-rabbit IgG (supplier: Jackson Immuno-Research)
Validation	HDAC1 (ABCAM ab19845) (https://www.abcam.com/hdac1-antibody-ab19845.html) : Reacts with: Mouse, Rat, Human, African green monkey / Synthetic peptide conjugated to KLH derived from within residues 450 to the C-terminus of Human HDAC1. / Relevant citation: e.g. PMID: 29302039. | HDAC2 (ABCAM ab16032) (https://www.abcam.com/hdac2-antibody-ab16032.html): Reacts with: Mouse, Rat, Human, African green monkey / Synthetic peptide conjugated to KLH derived from within residues 450 to the C-terminus of Human HDAC2. / Relevant citation: e.g. PMID: 29055107 | HDAC3 (ABCAM y415-ab32369) (https://www.abcam.com/hdac3-

antibody-y415-ab32369.html): Reacts with: Mouse, Rat, Human / Synthetic peptide within Human HDAC3 (C terminal). The exact sequence is proprietary. / Relevant citation : e.g. PMID: 29115379.

Animals and other organisms

Policy information about studies involving animals; ARRIVE guidelines recommended for reporting animal research

Laboratory animals	TgF344-AD rats (10-12 months old): Presenilin 1 (PS1ΔE9) and APP Swedish (KM670/671NL) mutations, and their respective control littermates (Fischer background). McGill-R-Thy1-APP rats (14-16 months old): Swedish double (K670N/M671L45) and the Indiana (V717F46) mutations, and their respective control littermates (Wistar). For both models, we used male and female rats and the groups were matched for sex.
Wild animals	The study did not involve wild animals.
Field-collected samples	The study did not involve samples collected from the field.
Ethics oversight	All rat work followed the National Institutes of Health guidelines and was approved by the McGill Animal Care Ethics Committee.

Note that full information on the approval of the study protocol must also be provided in the manuscript.

Human research participants

Policy information about studies involving human research participants

Population characteristics	A total of 94 participants aged 18 to 91 years were evaluated. Entire study population: Young CN = 25 (12 Male); Elderly CN = 28 (13 Male); MCI = 15 (9 Male); AD = 26 (17 Male). The demographics of the human subjects in each study site are described in Table 1.
Recruitment	The study participants were recruited for the present study from the community or outpatients at the McGill University Research Centre for Studies in Aging (MCSA) (Canada) and Massachusetts General Hospital (MGH) (United States) memory clinics. Therefore, the population recruited for this study represents a group of persons motivated to participate in a dementia-related study. As such, for reasons related to self-selection bias, these individuals may not entirely represent the general population. However, we do not believe that this had an impact on our results.
Ethics oversight	The human study was approved by the appropriate institutional ethics committees at the Douglas Mental Health University Institute Research Ethics Board, Montreal Neurological Instituted PET working committee, Partners HealthCare Institutional Review Board, and Massachusetts General Hospital Radioactive Drug Research Committee, and written informed consent was obtained from the participants.

Note that full information on the approval of the study protocol must also be provided in the manuscript.
